# Case Report: Behçet’s disease accompanied with vitiligo

**DOI:** 10.12688/f1000research.11025.1

**Published:** 2017-03-23

**Authors:** Ragıp Ertaş, Kemal Özyurt, Atıl Avcı, Sule Ketenci Ertas, Mustafa Atasoy

**Affiliations:** 1 Dermatology Department, Kayseri Training and Research Hospital, Kayseri, 38050, Turkey; 2 Department of Rheumatology, Erciyes University Faculty of Medicine, Kayseri, 38039, Turkey

**Keywords:** Behçet’s Disease, Vitiligo, Autoimmunity, Autoinflammatory, Depigmentation, Erythema nodosum, Thrombophlebitis, Arthritis

## Abstract

Recently, a few case reports and clinical studies have been published that explore the association of Behçet’s Disease (BD) and vitiligo, with conflicting results. Genetic and immunological properties of BD and presence of autoantibodies support autoimmunity, but clinical features suggest autoinflammatory diseases. BD is thought to be a cornerstone between autoimmune and autoinflammatory diseases. On the other hand, vitiligo has been accepted as an autoimmune disease with associations of other autoimmune disorders and there is a possible role of autoimmunity in pathogenesis of the disease. Significant advances have been made understanding the pathogenesis and genetics of BD. However, it is worth presenting rare clinical variants for improving the clinical understanding of BD. Herein, we are presenting a case with diagnosis of both Behçet’s disease and vitiligo in same patient, which is a rare occurrence. Discussion and demonstrating the association of these two diseases may give rise to understanding similar and different aspects of autoimmunity and autoinflammatory pathogenesis of both diseases.

## Introduction

Behçet's disease (BD) is a systemic disease with an unknown origin characterized by recurrent oral ulcers, mucocutaneus disorders and ocular findings. BD may be life-threatening, affecting the central nervous system, large vessels and the gastrointestinal tract ^1^. Numerous studies have investigated the etiopathogenesis of BD over a long period, but the etiology and mechanisms of pathogenesis have not yet been fully explained ^2^.

Vitiligo is a chronic depigmenting disorder representing white patches in the skin or hair extinct of functional melanocytes ^3^. Autoimmunity has been implicated in the pathogenesis of the disease, and associations with autoimmune diseases have been demonstrated ^4^.

Here, we present a unique case of BD and vitiligo in the same patient. This is a very rare condition and gives the opportunity to understand similar and different aspects of autoimmunity and autoinflammatory pathogenesis of both diseases by observing clinical and laboratory findings.

## Case report

A 24-year-old woman was admitted to the Clinic of Dermatology at the Kayseri Training and Research Hospital. The patient complained of swelling and pain in her legs for two weeks. Medical history of the patient included monthly relapsing oral aphthous ulcers for three years, and one attack of thrombophlebitis and arthritis previously. She had received treatment in various clinics and times for relapsing oral aphthous ulcers, including colchicum tablets, mouthwashes, corticosteroid and antibiotic creams. For thrombophlebitis and arthritis she was hospitalized and given therapy. The patient had vitiligo for 14 years. Her relatives had neither BD nor vitiligo.

**Figure 1.**
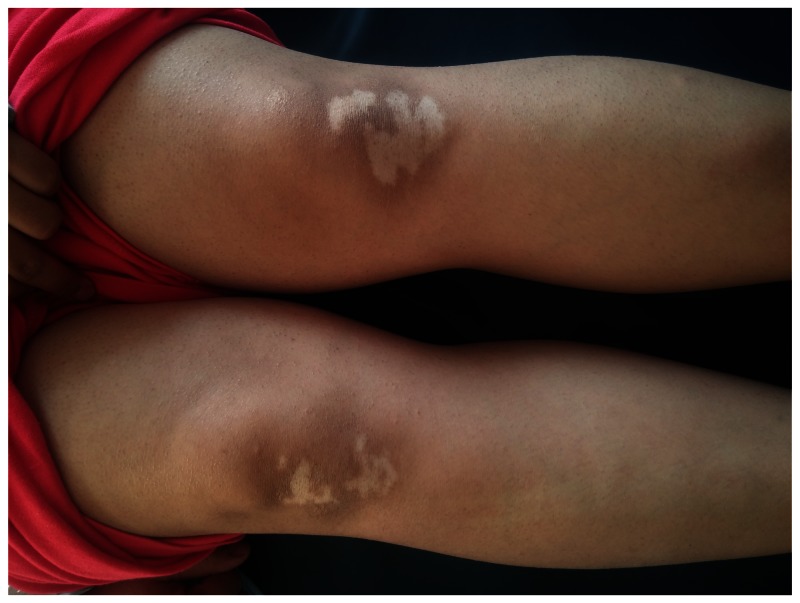
Depigmented patches on the bilateral knees.

A physical examination revealed erythema nodosum-like eruptions on the patient’s legs, and white, depigmented patches on the patient’s bilateral lateral malleolus, wrists, eyelids, knees, fingers and an oral aphthous ulcer on the lower lip mucosa ( Figure 1– Figure 4). An ophthalmological examination resulted in normal findings even though the patient had pain in her eyes. A pathergy test was negative. Laboratory examination showed hemoglobin,10.8 gr/dL(reference level,12–16gr/dL);platelet count,285 10^3/uL(130–400 10^3/uL);white cell count,635 10^3/uL (46–10210^3/uL);serum folic acid,4.84 ng/ml(3.1–17.54ng/ml);serum ferritin,8.5 ng/ml (110–305ng/ml);vitamin B12, 217 pg/ml(126-505pg/ml);serum iron,28 ug/dL (60–180ug/dL);serum total iron binding capacity,345 ug/dL (155–355ug/dL); C-reactive protein,5.11 mg/L (0–5mg/L);erythrocyte sedimentation rate,22 mm/h (0–20mm/h);rheumatoid factor,10.2 IU/ml (0–15IU/ml);serum antistreptolysin-o titer,174IU/ml (0–200IU/ml); free T3,3.68 pg/ml (2.5–3.9); free T4, 0.75 ng/dl (0.54–1.24 ng/dl); thyroid stimulating hormone,1.56 mIU/L (0.4–5.6mIU/L); antithyroglobulin antibody test,<2.2 IU/ml (0–4IU/ml); antithyroid peroxidase antibody test,0.6IU/ml (0–9IU/ml).

**Figure 2.**
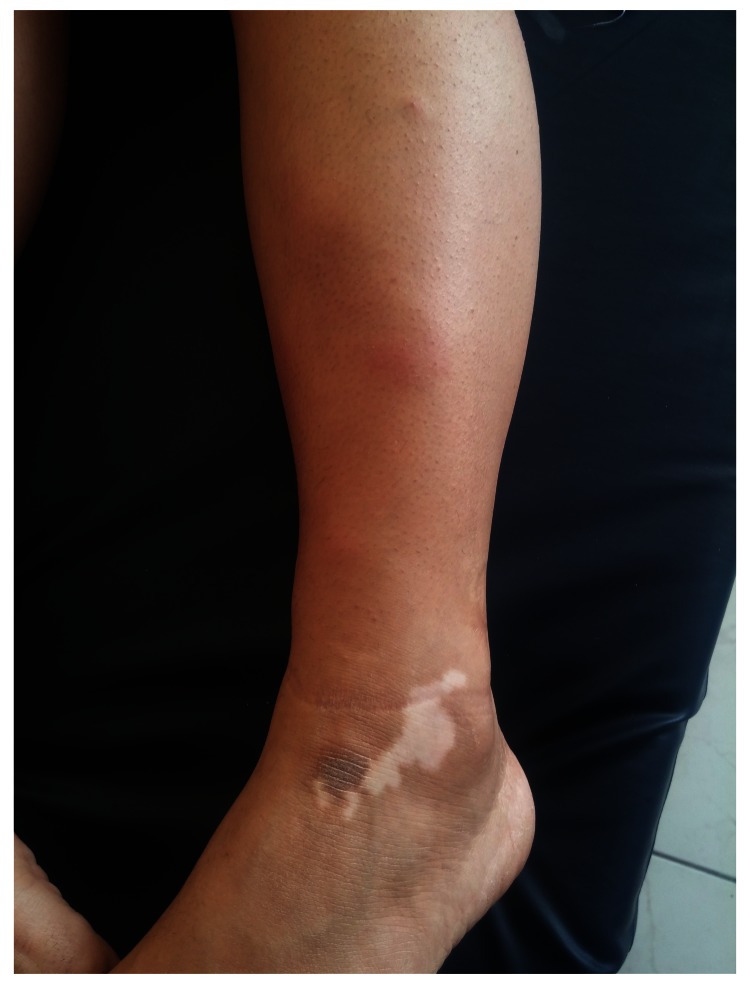
Erythema nodosum-like eruptions and depigmented patches on the lateral malleolus.

**Figure 3.**
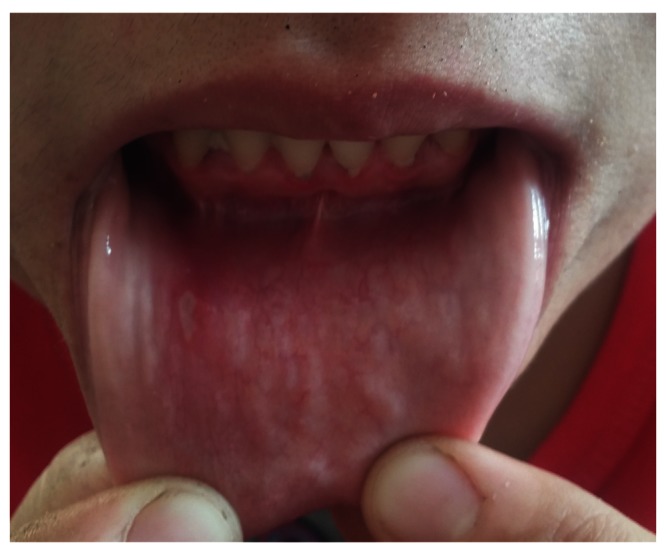
An aphthous ulcer on the lower lip mucosa.

**Figure 4.**
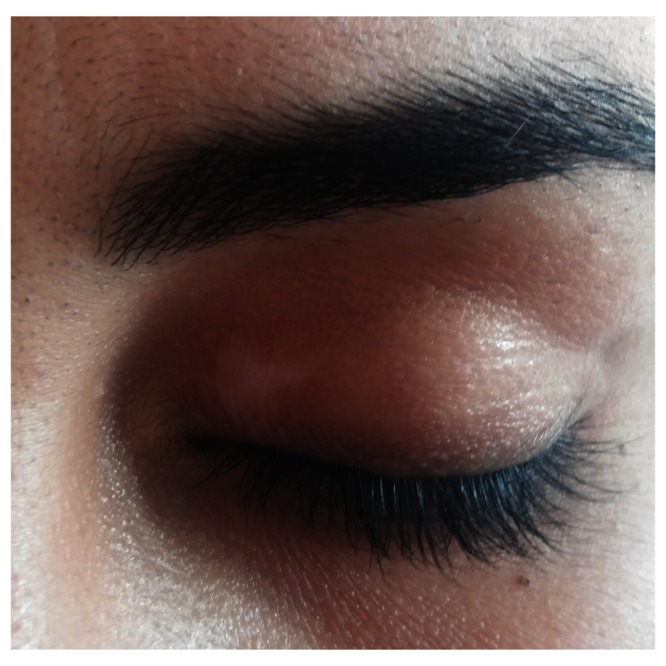
A depigmented patch on the upper eyelid.

A diagnosis of BD was made according to the International Criteria for Behçets Disease (ICBD) ^5^ and vitiligo was diagnosed based on prior physical examination. Diagnosis of BD, according to the ICBD, was based on only clinical features, but not any laboratory finding. For the ICBD, ocular lesions, oral aphthosis and genital aphthosis are each assigned 2 points, while skin lesions, central nervous system involvement and vascular manifestations are assigned 1 point each. The pathergy test was assigned 1 point. A patient scoring 4 points is classified as having BD. Our patient had 5 points: 2 for oral aphthosis, 1 for erythema nodosum and 1 for thrombophlebitis. Additionally, laboratory results mentioned above showed an iron deficiency anemia.

## Follow-up and outcomes

The patient was hospitalized and treated in our dermatology clinic for 10 days. She was given systemic corticosteroid and wet dressing for erythema nodosum-like eruptions on her legs. These lesions improved and she was discharged at the end of 10 days. She was not living in borders of our province and was recommended for follow-up in a local dermatology clinic.

## Discussion

Clinical and immunological understandings of the disease suggest BD is a cornerstone between autoimmune and inflammatory disease. Clinical features and male predominance suggest inflammatory diseases; however, sharing class I MHC association in genetic details and presence of autoantibodies in patients supports autoimmunity ^2^. Clinical characteristics and symptoms are the main factors for diagnosing BD, but a specific diagnostic feature or laboratory method is not yet available. The clinical features of patients in countries with a high prevalence of BD may help to clarify the pathogenesis of BD ^1^. Here we present a case of BD accompanied by vitiligo. Vitiligo is a common skin disorder and various factors participate in the etiopathogenesis, which causes autoimmune melanocytic destruction. Autoimmune thyroid diseases and pernicious anemia are frequently associated with vitiligo ^3, 4^. Recently, a few case reports and clinical studies have been published that demonstrate the association of BD with vitiligo, with conflicting results. Oran *et al*. showed that the frequency of vitiligo was not increased among patients with BD ^6^, while two different reports mentioned the coexistence of vitiligo and BD ^7, 8^. In addition, Guney *et al*. claimed that vitiligo occurred during interferon therapy in a patient with BD ^9^.

Vogt–Koyanagi–Harada (VKH) syndrome is an inflammatory disorder characterized by bilateral panuveitis, and is frequently associated with poliosis, vitiligo, alopecia, central nervous system and auditory symptoms ^10^. VKH syndrome is not often mistaken as BD. However, VKH syndrome has similar properties to BD and the etiology of both diseases remains unknown; however, an autoimmune response has been presumed to be implicated in their pathogenesis. Hu *et al*. mentioned TT genotype of rs7574865 in STAT4 gene may be a susceptible factor for VKH syndrome in a Chinese Han population, and GG genotype of this SNP may confer susceptibility in male BD patients ^11^. Our patient had only vitiligo and no other symptoms of VKH syndrome.

These case reports and studies give rise to thought about the association of BD and vitiligo. In our case, vitiligo had been present for 14 years before the diagnosis of BD. Antithyroid autoantibodies are not included in the diagnosis of BD, but show evidence of autoimmunity. These were negative in our patient. We don’t know whether a unique genetic predisposition or any environmental or infectious factor caused this status. Interestingly, Karincaoglu *et al.* declared incidental coexistence of BD and vitiligo and also koebnerization of genital ulceration of BD ^7^. However, in their case, the patient had vitiligo patches not only in the scar area of genital region, but also on other body surfaces.

Vitiligo may be only one symptom of a big picture, as in VKH syndrome ^12^. A different disease may have the features of BD and vitiligo. Indeed, all these implications are speculative and we need new studies and cases. We present a case of BD accompanied with vitiligo, a rare clinical variant of BD, which may help to improve the clinical understanding of BD.

## Consent

Written informed consent was obtained from the patient for the publication of the manuscript.
