# Supplementary material for: Case Report: Behçet’s disease accompanied with vitiligo
Source: F1000Res. 2017 Mar 23;6:310. [Version 1] doi: 10.12688/f1000research.11025.1 (PMC5428486; doi:10.12688/f1000research.11025.1)
Supplement: Peer review history

# Peer review: Referee response for version 1

**DOI:** 10.5256/f1000research.11892.r22530

**Published:** 2017-05-05

**Authors:** Rasheedunnisa Begum

**Affiliations:** 1 Department of Biochemistry, Faculty of Science, Maharaja Sayajirao University of Baroda, Vadodara, Gujurat, India

1. Authors should mention the onset of vitiligo and Behçet’s disease (BD) in clinical history. Moreover, it would be interesting if authors can mention the prevalence of vitiligo and BD for better correlation of the present study.
2. Vitiligo is a disorder without any gender biasness, as the subject recruited for the present study is a female it would add impact to the present study if authors should discuss the gender biasness for BD and discuss the same.
3. Authors should clearly state if depigmentation is due to vitiligo as there are many other depigmentation disorder. Was there any confirmation under wood’s lamp for the same?
4. In the discussion section authors have mentioned association of vitiligo with other autoimmune disorders, for the same they should cite recent reports with higher sample size (for e.g. autoimmunity in onset and progression of vitiligo where atypical autoimmune disorder Thyroid has been discussed and many more).
5. Authors should also mention the extent of depigmentation, type and the activity of vitiligo in the patient as per standard classification guidelines.
6. The findings of laboratory examination along with the normal range can be represented in a tabular form to make it clearer.
7. Authors should also mention whether Koebner phenomenon was observed in the patient or not and discuss the same.
8. Authors should also mention whether approval from respective ethics committee was obtained for publishing the case study.

I have read this submission. I believe that I have an appropriate level of expertise to confirm that it is of an acceptable scientific standard.

# Peer review: Referee response for version 1

**DOI:** 10.5256/f1000research.11892.r21204

**Published:** 2017-04-18

**Authors:** Zafer Türkoğlu

**Affiliations:** 1 Department of Dermatology, School of Medicine, Goztepe Training and Research Hospital, Istanbul Medeniyet University, Istanbul, Turkey

- The case report that is being reported has been carried out well with no flaws in the design or methodology.
- The case report was reported correctly, with acknowledgement of the existing body of work.
- The work provides sufficient details for it to be useful for other practitioners.

It is suitable for indexing.

I have read this submission. I believe that I have an appropriate level of expertise to confirm that it is of an acceptable scientific standard.
